# Efficacy and safety of Kunxinning granules in patients with menopausal syndrome: a multicenter, randomized, double-blinded, and placebo-controlled trial

**DOI:** 10.3389/fphar.2025.1512110

**Published:** 2025-07-10

**Authors:** Guirong Zhou, Danfei Chen, Lin Qian, Dianrong Song, Qin Zhang, Ying Yan, Jie Lin, Li Li, Guiping Wan, Shuping Li, Lili Hou, Yi He, Cong Qi, Weian Yuan

**Affiliations:** ^1^ National Key Laboratory of Chinese Medicine Modernization, Tasly Pharmaceutical Group Co., Ltd., Tianjin, China; ^2^ Zhejiang Institute of Traditional Chinese Medicine Co., Ltd., Hangzhou, China; ^3^ Department of Acupuncture and Moxibustion, Shuguang Hospital Affiliated to Shanghai University of Traditional Chinese Medicine, Shanghai, China; ^4^ Department of Internal Medicine (TCM), The Second Affiliated Hospital of Tianjin University of Traditional Chinese Medicine, Tianjin, China; ^5^ Department of Orthopedics and Traumatology (TCM), Guangxing Hospital Affiliated to Zhejiang University of Traditional Chinese Medicine, Hangzhou, China; ^6^ Department of Spleen and Stomach Diseases (TCM), The First Affiliated Hospital of Tianjin University of Traditional Chinese Medicine, Tianjin, China; ^7^ Department of Gynecology (TCM) , The First Affiliated Hospital of Hunan University of Traditional Chinese Medicine, Changsha, China; ^8^ Department of Geriatrics (TCM), Nanjing Hospital of Traditional Chinese Medicine, Nanjing, China; ^9^ Department of Integrated Traditional Chinese and Western Medicine, Jiangsu Provincial Hospital of Integrated Traditional Chinese and Western Medicine, Nanjing, China; ^10^ Department of Pediatrics (TCM), Changzhou Traditional Chinese Medicine Hospital, Changzhou, China; ^11^ Department of Obstetrics and Gynecology, Nanjing Maternal and Child Health Hospital, Nanjing, China

**Keywords:** full analysis set, Kunxinning granules, menopausal syndrome, modified ADR, adverse drug reactions, adverse events (AE), confidence interval (CI), full analysis set (FAS), follicle-stimulating hormone (FSH)

## Abstract

**Background:**

Menopausal syndrome is a general term for a series of physical and psychological symptoms that women experience during menopause and perimenopause, which can lead to the occurrence of a variety of diseases. Many ingredients in Kunxinning granules have been used clinically to treat menopausal syndrome. However, evidence for its effectiveness is lacking.

**Purpose:**

To investigate the efficacy and safety of Kunxinning Granules in patients with menopausal syndrome and to fully verify its clinical application value. Study design: A multicenter, randomized, double-blinded, placebo-controlled trial.

**Methods:**

Eligible participants from 9 hospitals were randomly assigned in a ratio of 3:1 to the experimental group (Kunxinning granules) and the placebo group (Kunxinning granules simulated agent) for 12 weeks. The primary efficacy outcome was the score reduction of the modified Kupperman index compared with the baseline. The secondary efficacy index is the disappearance rate of 13 single symptoms of the modified Kupperman index. The evaluation time points are baseline, 4 weeks, 8 weeks, 12 weeks and 4 weeks of drug withdrawal.

**Results:**

A total of 475 patients (356 in experimental group and 119 in placebo groups) were included in the analysis. The modified Kupperman index of patients in the experimental group and placebo group were 28.81 ± 6.81 and 27.61 ± 7.38. The modified Kupperman index of the experimental group improved after 4 weeks of treatment (experimental group: 21.45 ± 6.29, placebo group: 23.79 ± 6.90, 97.5%CI: -3.68 to −0.99, *p* = 0.007) and 8 weeks (experimental group: 15.18 ± 5.82, placebo group: 20.93 ± 7.29, 97.5%CI: −7.05 to −4.45, *p* < 0.0001), 12 weeks (experimental group: 10.27 ± 5.44, placebo group: 19.70 ± 7.25, 97.5%CI: −10.68 to −8.19, *p* < 0.0001) and 4 weeks of drug withdrawal (experimental group: 10.27 ± 5.44, placebo group: 19.70 ± 7.25, 97.5%CI: −10.86 to −8.35, *p* < 0.0001) were significantly lower than placebo group. Moreover, the experimental group’s modified Kupperman index reduction from baseline was better than that of the placebo group at 4 weeks, 8 weeks, 12 weeks of treatment and 4 weeks of drug withdrawal (*p* < 0.0001). Moreover, the experimental group had a good improvement effect on all 13 symptoms of the modified Kupperman index after 12 weeks of treatment. There were no significant differences in adverse events between the two groups.

**Conclusion:**

Kunxinning granules are a promising treatment for menopausal syndrome which can effectively reduce patients’ hot flashes and sweating, insomnia, irritability and urinary symptoms and improve their quality of life.

**Clinical Trial Registration:**

http://www.chinadrugtrials.org.cn/clinicaltrials.searchlist.dhtml, identifier CTR20140289.

## 1 Introduction

Menopausal syndrome (Avis et al., 2001) is a series of physical and mental symptoms caused by fluctuations or declines in hormone levels in women before and after menopause, which is one of the common chronic diseases in women. As the world’s population ages and life expectancy increases, the incidence of menopausal syndrome in women around the world is gradually increasing that has a serious impact on women’s physical and mental health (Chen and Chen, 2021; Li et al., 2023). The subjective symptoms of patients with menopausal syndrome mainly include hot flashes, anxiety, sleep disorders, vaginal atrophy and muscle pain (Santoro et al., 2015). Menopausal syndrome not only affects women’s quality of life (Angelou et al., 2020), but also significantly increases the probability of multiple diseases, such as cardiovascular disease (Nappi and Simoncini, 2021), osteoporosis (Mohapatra et al., 2024), metabolic syndrome (Medina-Contreras et al., 2020) and diabetes (Lambrinoudaki et al., 2022).

The treatment of menopausal syndrome aims to relieve symptoms and improve patients’ quality of life (Palacios et al., 2020). Common treatments include mind-body therapy (Shorey et al., 2020), hormone replacement therapy (HRT) (Vigneswaran and Hamoda, 2022) and non-hormone drug therapy (Pan et al., 2023). However, there are differences in symptoms among female patients with menopausal syndrome, menopausal syndrome involves multiple systems, and the symptoms are also very diverse, such as physical, psychological and social problems (Wang and Yu, 2021). Currently, the most commonly used treatment is HRT (Langer et al., 2021). HRT is very effective in relieving menopausal symptoms, but its long-term use may increase the risk of breast cancer, cardiovascular disease and stroke (Genazzani et al., 2021). Moreover, HRT has strict indications, contraindications and cautions (Lindberg et al., 2020). Patients with long-term use require regular comprehensive physical examinations, and it is not suitable for all patients with menopausal syndrome.

There is no single best treatment for menopausal syndrome, and the effectiveness and safety of various treatments remain controversial (Pandozzi et al., 2022). Natural medicines for the treatment of menopausal syndrome are gaining more and more attention with many patients looking to relieve symptoms through non-hormonal methods (Wang and Yu, 2021; Lee et al., 2020). The isoflavones in soy extract have been shown to be widely used in postmenopausal patients as an alternative treatment for menopause-related symptoms (Chen and Chen, 2021). Studies have found that isoflavones can reduce the risk of coronary heart disease, breast cancer and colorectal cancer in patients with menopausal syndrome and can effectively improve the patients’ quality of life (Barańska et al., 2021). Black cohosh can relieve menopausal symptoms by affecting neurotransmitters and hormone levels in the body (Hedaoo et al., 2024). Study found that menopausal women using black cohosh experienced relief from hot flashes, night sweats and mood swings (Mohapatra et al., 2022). And low doses of black cohosh can effectively improve menopausal symptoms without any adverse effects (Bai et al., 2007).

Kunxinning granules are a combination of various natural species, including *Rehmannia glutinosa*, *Astragalus membranaceus*, *Curculigo orchioides*, *Epimedium brevicornu, Paeonia lactiflora*, *Haliotis diversicolor* and *Albizzia julibrissin* (Jiancheng et al., 2005). Many of these substances have been found to be useful in treating menopausal syndrome (Portella et al., 2024; Zhou et al., 2021). *Rehmannia glutinosa* has the effect of replenishing blood which can help relieve yin deficiency symptoms such as hot flashes, night sweats, and dry mouth in menopausal women (Kim and Kim, 2022). Moreover, *R. glutinosa* is effective against thermal illnesses and can help improve irritability caused by menopause. *Astragalus membranaceus* can help relieve symptoms of dizziness, tinnitus, and have certain effects on mood swings and insomnia in menopausal women (Lin et al., 2020). In order to explore the efficacy and safety of Kunxinning granules in treating menopausal syndrome, we conducted a multicenter, randomized, double-blinded and placebo controlled trial.

## 2 Methods

### 2.1 Study design

Kunxinning Granules is an innovative Chinese medicine for the treatment of female menopausal syndrome approved by the National Medical Products Administration (NMPA) in 2021 (approval number: National Medicine Standard Z20210006). Kunxinning Granules is a mixture of different metabolites, including *R. glutinosa*, *A. membranaceus*, *C. orchioides*, *E. brevicornu, P. lactiflora*, *H. diversicolor* and *Albizzia julibrissin.* We first conducted three different (orthogonal) fingerprint analyses on Kunxinning Granules (Supplementary Figure S1) and we found a total of 115 chemical components and identified 109 compounds, including 13 phenolic components, 6 lignan components, 43 flavonoid components, 7 triterpenoid saponins, 25 monoterpenoid components, 11 phenylpropanoid components and 4 other components (Supplementary Table S1). Each 6 g of Kunxinning Granules has 1.5 g of dried root tuber of *R. glutinosa* (Gaertn.) DC. [Orobanchaceae; Rehmanniae radix praeparata], 1.5 g of dried root of *A. membranaceus* (Fisch.) Bunge [Fabaceae; Astragali radix], 1.2 g of dried rhizome of *C. orchioides* Gaertn. [Hypoxidaceae; Curculiginis rhizoma], 1.0 g of dried leaf of *E. brevicornu* Maxim. [Berberidaceae; Epimedii folium], 0.7 g of dried root of *P. lactiflora* Pall. [Paeoniaceae; Paeoniae radix alba], 0.6 g of shell of *H. diversicolor* Reeve [Haliotidae; Haliotidis concha], 0.5 g of dried bark of *Albizia julibrissin* Durazz. [Fabaceae; Albiziae cortex], the rest is auxiliary materials. All Chinese medicinal materials were identified by qualified experts in botanical drugs using morphological methods. The identification work was completed by Professor Cong Qi (affiliation: Zhejiang Institute of Traditional Chinese Medicine). The voucher specimens of each medicinal material have been stored in the specimen room of Zhejiang Institute of Traditional Chinese Medicine. The samples used in this clinical study were produced in strict accordance with product process standards and under GMP management. The materials used include decoction pieces, excipients, preparations and packaging materials. The decoction pieces comply with the standards of Part I of the Chinese Pharmacopoeia, the excipients and packaging materials comply with the standards of Part IV of the Chinese Pharmacopoeia, and the preparation standards meet the quality standards of marketed products approved by NMPA and have been verified and confirmed by the Food and Drug Review and Inspection Center of the National Medical Products Administration.

Through a multi-center, randomized, double-blind, placebo-controlled trial, the effect of Kunxinning granules on relieving related symptoms and improving the quality of life of female patients with menopausal syndrome was evaluated. Patient representatives participated in the design and implementation of the study. At the beginning of the study, the research team communicated with patient representatives to solicit their opinions on research questions and research methods. The research results were also published on a public platform to provide feedback to relevant groups. In order to ensure the rights and interests of the subjects and the authenticity and reliability of the research results, the supervisors will work with the heads of each trial center to train the researchers on the trial protocol before the start of the clinical trial. In addition, the monitors conduct consistency tests on the quantification standards for symptoms and signs so that researchers can understand and become familiar with the nature, effects, efficacy and safety of the experimental drugs. The sponsor appoints monitors with medical background to monitor the entire trial process. The trial was registered with the China Clinical Trial Registration Center (http://www.chinadrugtrials.org.cn/clinicaltrials.searchlist.dhtml) in April 2014 (registration number: CTR20140289), and CONSORT (Consolidated Standards of Reporting Trials) and GCP (Good Clinical Practice) were followed during the experiment.

### 2.2 Participants

The subjects of this study were female patients with menopausal syndrome from 9 hospitals from 2014-04-04 to 2015-10-27, namely, Shuguang Hospital Affiliated to Shanghai University of Traditional Chinese Medicine, the Second Affiliated Hospital of Tianjin University of Traditional Chinese Medicine, Guangxing Hospital Affiliated to Zhejiang University of Traditional Chinese Medicine, Tianjin The First Affiliated Hospital of University of Chinese Medicine, the First Affiliated Hospital of Hunan University of Chinese Medicine, Nanjing Hospital of Traditional Chinese Medicine, Jiangsu Hospital of Integrated Traditional Chinese and Western Medicine, Changzhou Hospital of Traditional Chinese Medicine, and Nanjing Maternal and Child Health Hospital. The screening criteria for subjects in this study are: 1) women aged 45-55 years old; 2) female patients with menopausal syndrome who meet Western medical diagnostic standards (Lumsden et al., 2016); 3) menstrual disorders or menopause for more than 3 months; 4) the patient’s modified Kupperman index (Tao et al., 2013) is ≥ 16 points; 5) follicle-stimulating hormone (FSH) > 10U/L. The exclusion criteria for subjects are: 1) Malignant uterine tumors, uterine fibroids > 2cm, endometrial polyps, endometrial thickness ≥0.5 cm in postmenopausal women; 2) malignant breast tumors and severe breast hyperplasia; 3) hypertension; coronary atherosclerotic heart disease; angina pectoris; abnormal electrocardiogram; abnormal liver and kidney function; thyroid disease (such as hyperthyroidism, goiter); pheochromocytoma; hematopoietic system, liver and kidney and severe disease of internal organs; 4) neurasthenia and mental illness (such as patients with depression and anxiety disorders); 5) disabled patients; 6) those who are allergic to this medicine; 7) people with frailty and other complicated diseases; 8) those who are using the same type of traditional Chinese medicine or hormone therapy.

All drugs and treatments that may have cross-effects with the research intervention were prohibited during this study. All participants were strictly monitored and recorded by researchers before enrollment, during the intervention and follow-up stages to ensure the independence of the research intervention and the reliability of the results. All subjects in this study signed informed consent, and the trial protocol was approved by the ethics committee of each hospital research center (Ethics approval number: 2007L05109) and conformed to the Declaration of Helsinki.

### 2.3 Randomization, allocation concealment and blinding

In this study, eligible female patients with menopausal syndrome were selected and randomly assigned to the experimental group (Kunxinning granules) and the placebo group (Kunxinning granule simulator) in a ratio of 3:1 (360:120) for treatment. The placebo group’s patient drug was cornstarch. SAS 9.4 software (SAS Institute Inc., Cary, United States) (Guide, 2012) was used to generate random numbers for all patients, and these patients were randomly divided into 2 groups according to proportion. The random allocation sequence was stored in a password-protected database before the start of the study to ensure allocation concealment and was not accessible to research site staff. After each subject was enrolled, the study coordinator obtained their allocation group through the interactive web response system. Kunxinning granules and Kunxinning granule simulator are both produced and inspected by Zhejiang Institute of Traditional Chinese Medicine Co., Ltd., and the specifications are 6 g per bag. The dosage is 1 bag each time, 3 times a day, orally after meals for 12 consecutive weeks. The two groups of drugs are consistent in terms of color, properties, odor, specifications, packaging, labels and markings to ensure blind implementation of clinical trials. The drugs in the experimental group and the control group all use the same packaging bags, and the drugs are distributed to the subjects according to the random numbers generated by SAS. All relevant researchers and subjects were blinded to drug allocation, and the numbers of the experimental and placebo groups were kept strictly confidential by the principal investigator.

### 2.4 Efficacy and safety assessment

Before administration, the Kupperman index was performed on all menopausal syndrome patients in the experimental group and the placebo group. After 4 weeks of treatment, 8 weeks of treatment, 12 weeks of treatment and 4 weeks of drug withdrawal, the Kupperman index of the patients in the experimental group and the placebo group were measured again. The modified Kupperman index is the Kupperman index of the experimental group and the placebo group after 4 weeks of treatment, 8 weeks of treatment, 12 weeks of treatment and 4 weeks of withdrawal minus the Kupperman index of the experimental group and the placebo group before treatment, which is the primary efficacy indicator. The secondary efficacy indicator is the score of 13 individual symptoms (Hot flashes and sweating, abnormal sensation, insomnia, irritability, urinary symptoms, painful intercourse (dyspareunia), depression, dizziness, fatigue, bone joint muscle pain, headache, palpitations formication). In addition, adverse events were assessed and recorded throughout the trial. Adverse events (AE) refer to all adverse medical events that occur after subjects receive experimental drugs, following the principles of GCP (National Medical Products Administration of China, 2020).

### 2.5 Statistical analysis

All statistical analyzes in this study were completed using SAS 9.4 software. This study used multiple imputation (MI) to handle the missing data. The imputation process built a model based on variables such as the patient’s baseline characteristics, historical scores and treatment groups to generate multiple complete data sets, and the final analysis results took the weighted average of the analysis results of these data sets. Patients who received at least one treatment and provided valid data were included in the full analysis set (FAS). The FAS data set is mainly used to evaluate the primary efficacy outcome and secondary efficacy effect. The per protocol pet set (PPS) set is a data set of participants who meet the requirements of the research protocol, excluding those who do not perform in accordance with the requirements of the research protocol, such as not taking medications on time and not following trial procedures. The safety evaluation used the safety set (SS) which is a data set for evaluating the safety of treatments. SS are data for all participants who received treatment and had at least one safety assessment.

Quantitative indicators consistent with a normal distribution were analyzed using the t-test, with tests for homogeneity of variances between groups conducted at a significance level of 0.05. According to the study of Tao et al. (2013), we expect the average difference in Kupperman index scores between the experimental drug group and the placebo group after intervention to be 10, with a standard deviation of 5. Under the conditions of setting the significance level α = 0.05 (two-sided) and the test power (1-β) = 90%, the calculation results show that at least 6 subjects should be included in each group. The Satterthwaite method was applied for correction when variances were unequal. For non-normally distributed indicators, the Wilcoxon rank-sum test and the Wilcoxon signed-rank test were used. Qualitative indicators are described as percentages, and quantitative indicators are presented as mean values and standard deviations (mean ± standard deviation (SD)). Hypothesis testing employed two-sided tests uniformly, providing test statistics and corresponding P-values, and calculating two-sided 95% confidence intervals (CIs). The change in Kupperman index score at 12 weeks of treatment and 4 weeks of drug withdrawal was predefined as the coprimary endpoint, α = 0.025 was set for each endpoint and 97.5% confidence intervals were reported accordingly. The results of the other time points (4 weeks of treatment and 8 weeks of treatment) were not adjusted for multiplicity. We have modified the statistical analysis in the methods. This study also used analysis of covariance (ANCOVA) to analyze the treatment effects at multiple time points. Statistical significance was defined as p ≤ 0.05, with higher significance at *p* ≤ 0.01.

## 3 Result

### 3.1 General characteristics of patients

**FIGURE 1:**
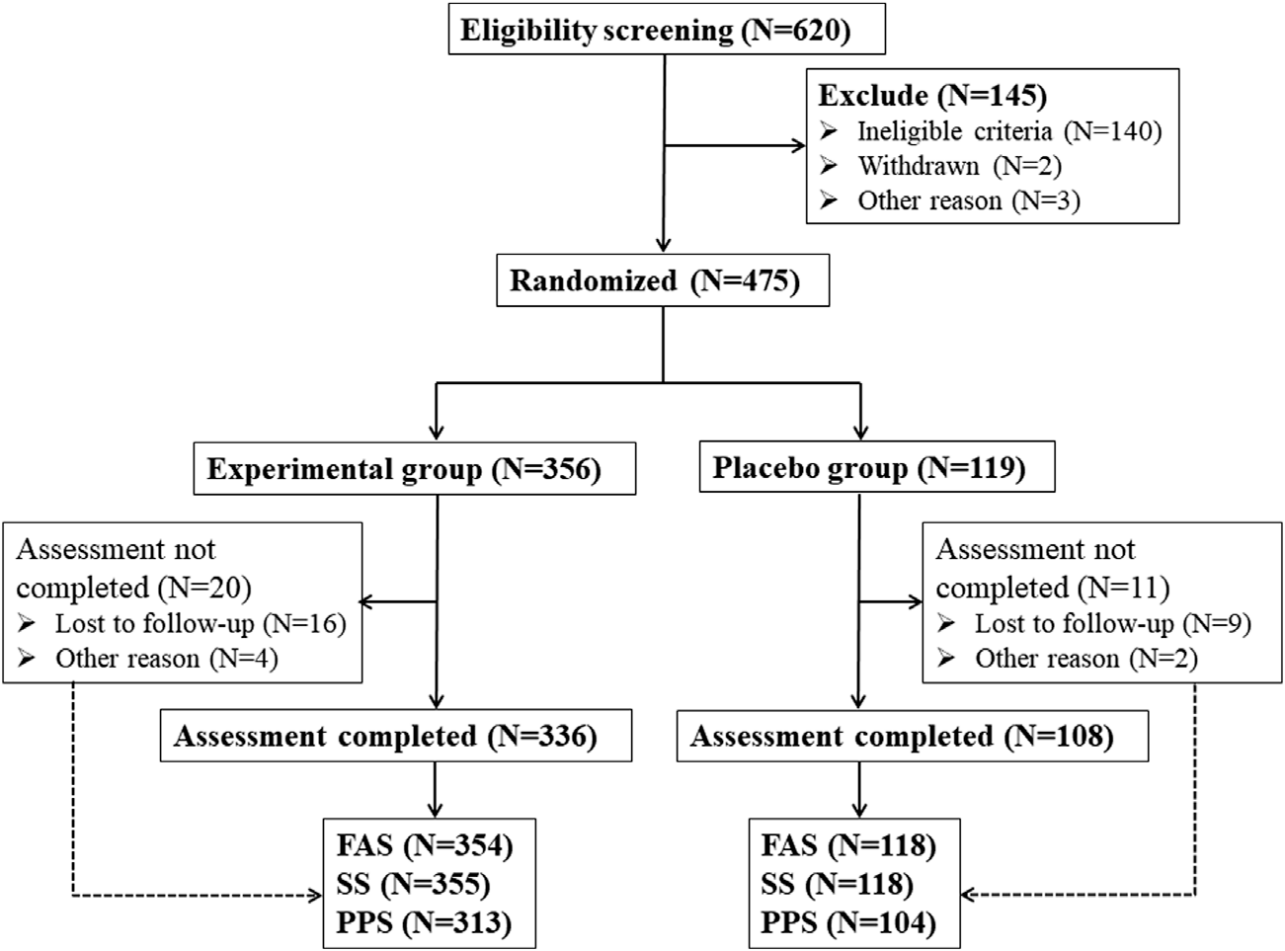
Schematic flowchart of clinical trial. Note: FAS (Full analysis set), SS (Safety set), PPS (Per protocol pet).

In this study, a total of 620 eligible patients who met the screening qualifications were obtained from 9 hospitals, and 145 patients met the exclusion criteria (Figure 1). 475 patients were randomly assigned to receive Kunxinning granule treatment (experimental group, N = 356) or placebo (placebo group, N = 119). In the experimental group, 336 (94.38%) patients completed all trial participation processes, and 20 (5.62%) patients were withdrawn from the trial due to loss of follow-up and other reasons. In the placebo group, 108 patients (90.75%) completed all trial participation processes, while 11 patients (9.25%) dropped out of the trial due to loss to follow-up and other reasons. Statistical analysis was conducted on the subject data of the experimental group and the placebo group, and the study found that there were 472 cases of FAS (354 cases in the experimental group and 118 cases in the placebo group). There were 473 cases in the SS group (355 cases in the experimental group and 118 cases in the placebo group). 58 patients were excluded from PPS due to various reasons, such as adverse events, other drug treatments, and unwillingness to continue treatment. Finally, 417 patients were included in PPS (313 patients in the experimental group and 104 patients in the placebo group).

This study further analyzed the baseline basic information of patients in the experimental group (n = 354) and placebo group (n = 118) (Table 1). The average age, course of disease, breaths, pulse, systolic blood pressure, diastolic blood pressure, resting heart rate, previous western medical treatment, previous TCM treatment, history of drug allergy, abnormal gynecological examination, endometrial thickness and estradiol of patients in experimental group, were 50.89 ± 3.1, 29.15 ± 19.66, 18.66 ± 1.32, 71.73 ± 7.49, 117.44 ± 8.86, 73.65 ± 6.22, 71.38 ± 7.48, 13(3.67%), 18(5.08%), 16(4.52%), 5(1.41%), 0.21 ± 0.11, 36.26 ± 59.79. And the average age, course of disease, breaths, pulse, systolic blood pressure, diastolic blood pressure, resting heart rate, previous western medical treatment, previous TCM treatment, history of drug allergy, abnormal gynecological examination, endometrial thickness and estradiol of patients in Placebo group were 50.68 ± 3.10, 30.87 ± 17.92, 18.66 ± 1.31, 73.66 ± 7.45, 116.89 ± 7.68, 74.13 ± 5.65, 73.61 ± 7.58, 3(2.54%), 11(9.32%), 4(3.42%), 3(2.54%), 0.23 ± 0.13, 63.45 ± 183.42. In addition, we also analyzed the incidence of 13 individual symptoms of menopausal syndrome in patients in the experimental group and Placebo group, and the study found that there was no significant difference in the incidence of 13 individual symptoms of menopausal syndrome in the experimental group and Placebo group.

**TABLE 1:** Baseline demographic and clinical characteristic of patients including in the FAS.

Characteristics	Experimental group (n = 354)	Placebo group (n = 118)	*p* value
Age (year) (mean ± SD)	50.89 ± 3.10	50.68 ± 3.10	0.5321
Course of disease (months) (mean ± SD)	29.15 ± 19.66	30.87 ± 17.92	0.3992
Breaths (times/minute) (mean ± SD)	18.66 ± 1.32	18.66 ± 1.31	1.0000
Pulse (times/minute) (mean ± SD)	71.73 ± 7.49	73.66 ± 7.45	0.0153*
Systolic blood pressure (mmHg) (mean ± SD)	117.44 ± 8.86	116.89 ± 7.68	0.5483
Diastolic blood pressure (mmHg) (mean ± SD)	73.65 ± 6.22	74.13 ± 5.65	0.4631
Resting heart rate (times/minute) (mean ± SD)	71.38 ± 7.48	73.61 ± 7.58	0.0553
Previous Western medical treatment (%)	13 (3.67)	3 (2.54)	0.5585
Previous TCM treatment (%)	18 (5.08)	11 (9.32)	0.0847
History of drug allergy (%)	16 (4.52)	4 (3.42)	0.5946
Abnormal gynecological examination (%)	5 (1.41)	3 (2.54)	0.3943
Endometrial thickness (cm) (mean ± SD)	0.21 ± 0.11	0.23 ± 0.13	0.2939
Estradiol (mean ± SD)	36.26 ± 59.79	63.45 ± 183.42	0.3845
Follicle stimulating hormone (mean ± SD)	57.66 ± 30.81	59.45 ± 34.21	0.5678
Luteinizing hormone (mean ± SD)	30.28 ± 14.58	31.95 ± 16.92	0.1259
Modified Kupperman index (mean ± SD)	28.81 ± 6.81	27.61 ± 7.38	0.1059
Hot flashes and sweating (%)	354 (100.0)	118 (100.0)	0.1006
Abnormal sensation (%)	309 (87.3)	103 (87.3)	0.8240
Insomnia (%)	343 (96.9)	115 (97.5)	0.9712
Irritability (%)	347 (98.0)	115 (97.5)	0.3931
Urinary symptoms (%)	226 (63.8)	76 (64.4)	0.8215
Painful intercourse (dyspareunia) (%)	210 (59.3)	69 (58.5)	0.6613
Depression (%)	241 (68.1)	77 (65.2)	0.5666
Dizziness (%)	291 (82.2)	94 (79.7)	0.6511
Fatigue (%)	337 (95.2)	107 (90.7)	0.1093
Bone joint muscle pain (%)	309 (87.3)	103 (87.3)	0.1867
Headache (%)	263 (74.3)	86 (72.9)	0.6526
Palpitations (%)	280 (79.1)	92 (78.0)	0.8250
Formication (%)	212 (59.9)	67 (56.8)	0.4379

Abbreviations: FAS, full analysis set; SD, standard deviation. * represents *p* < 0.05.

Except for pulse, the significance analysis results showed that there was no significant difference in these characteristics between patients in the experimental group and the placebo group. This shows that the two groups of patients are similar in this basic information, which allows subsequent research and analysis to be conducted.

### 3.2 Primary outcome

In order to explore the treatment effect of Kunxinning granules on female patients with menopausal syndrome, we conducted modified Kupperman indexs on patients in the experimental group and placebo group at baseline, 4 weeks of treatment, 8 weeks of treatment, 12 weeks of treatment and 4 weeks of drug withdrawal (Table 2). The modified Kupperman index of patients in the experimental group at baseline, 4 weeks of treatment, 8 weeks of treatment, 12 weeks of treatment and 4 weeks of drug withdrawal were 28.81 ± 6.81, 21.45 ± 6.29, 15.18 ± 5.82, 10.27 ± 5.44, 10.95 ± 5.61, while placebo group is 27.61 ± 7.38, 23.79 ± 6.90, 20.93 ± 7.29, 19.70 ± 7.25, 20.56 ± 7.07. The 95% confidence intervals at baseline, 4 weeks on treatment, 8 weeks on treatment, 12 weeks on treatment and 4 weeks of drug withdrawal were −0.25 to 2.65, −3.68 to −0.99, −7.05 to −4.45, −10.68 to −8.19, −10.86 to −8.35. At 4 weeks of treatment, 8 weeks of treatment, 12 weeks of treatment and 4 weeks of drug withdrawal, the modified Kupperman index of patients in the experimental group was significantly lower than that of the placebo group (p < 0.001).

**TABLE 2:** Modified Kupperman index of patients before and after treatment in the FAS.

Time	Experimental group (n = 354)	Placebo group (n = 118)	MD (95% CI)	*p* value
Baseline	28.81 ± 6.81	27.61 ± 7.38	1.20 (-0.25,2.65)	-
4 weeks of treatment (mean ± SD)	21.45 ± 6.29	23.79 ± 6.90	−2.34 (−3.68, −0.99)	0.0007***
8 weeks of treatment (mean ± SD)	15.18 ± 5.82	20.93 ± 7.29	−5.75 (−7.05, −4.45)	<0.0001***
12 weeks of treatment (mean ± SD)	10.27 ± 5.44	19.70 ± 7.25	−9.44 (−10.68, −8.19)	<0.0001***
4 weeks of drug withdrawal (mean ± SD)	10.95 ± 5.61	20.56 ± 7.07	−9.61 (−10.86, −8.35)	<0.0001***

Abbreviations: FAS, full analysis set; SD, standard deviation; MD, mean difference; CI, confidence interval. * represents *p* < 0.001.

**FIGURE 2:**
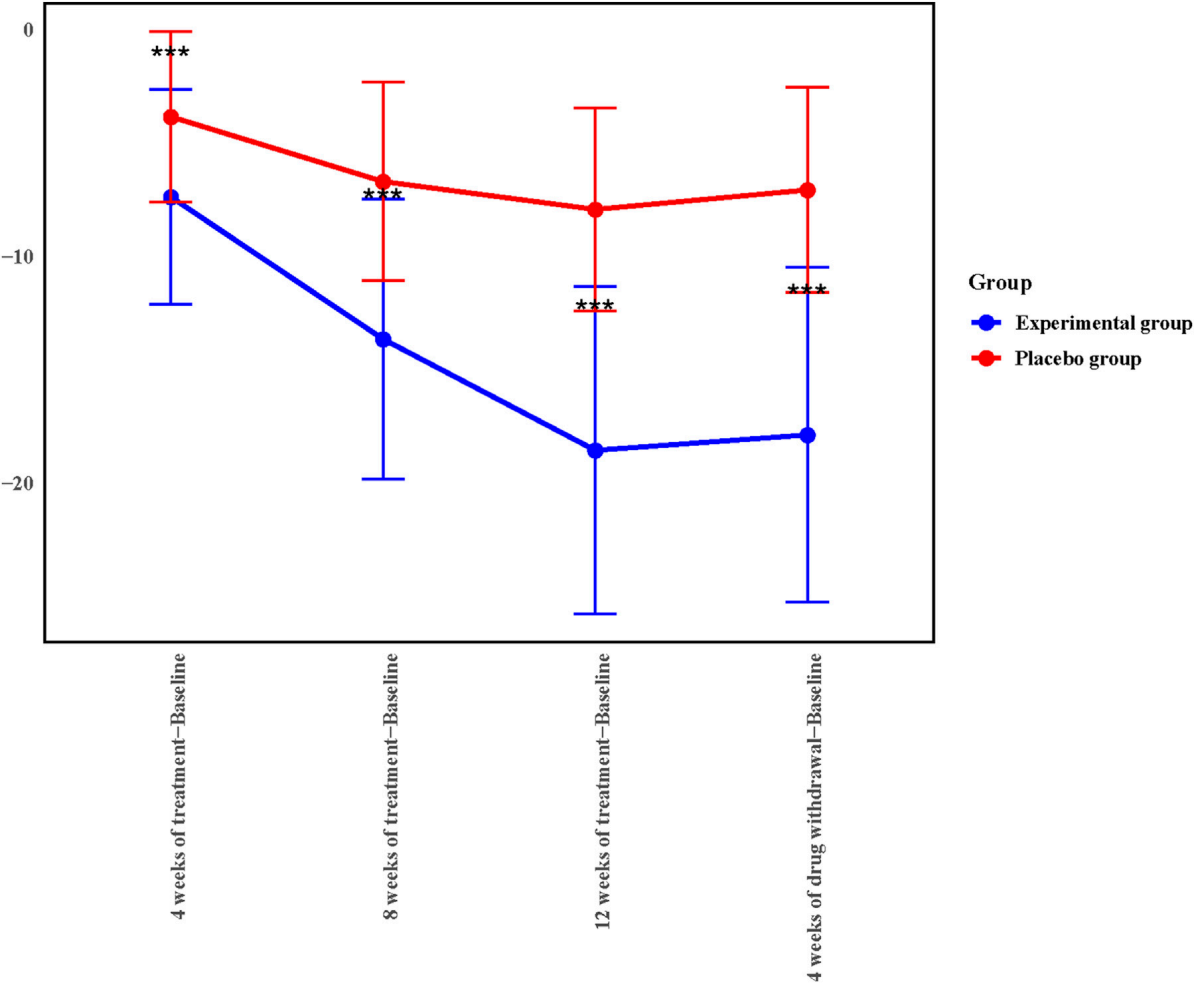
4, 8, 12 weeks of treatment, 4 weeks of drug withdrawal for the decrease in modified Kupperman index from baseline in experimental group and placebo group in the Full analysis set (FAS).

To further explore the therapeutic effect of Kunxinning granules on female patients with menopausal syndrome, we calculated the decrease in modified Kupperman index compared with the baseline after 4 weeks of treatment, 8 weeks of treatment, 12 weeks of treatment and 4 weeks of drug withdrawal (Figure 2). Compared with baseline, the decreases in modified Kupperman indexs of the experimental group at 4 weeks of treatment, 8 weeks of treatment, 12 weeks of treatment and 4 of drug withdrawal were −7.36 ± 4.73, −13.63 ± 6.19, −18.54 ± 7.22 and 17.86 ± 7.39 respectively. The decline values of the modified Kupperman index of the placebo group were −3.82 ± 3.75, −6.68 ± 4.38, −7.91 ± 4.49, and −7.05 ± 4.52 respectively. Correlation analysis results showed that the decrease in modified Kupperman index in experimental group and placebo group at different treatment stages compared with baseline was significantly correlated (*p* < 0.0001) (Supplementary Table S2). During the drug administration phase, the modified Kupperman indexs of patients in the experimental group were significantly reduced. Of drug withdrawal, the modified Kupperman indexs of the experimental group and placebo group increased. This demonstrates the therapeutic effect of Kunxinning granules on female patients with menopausal syndrome.

### 3.3 Secondary outcomes

This study further conducted a statistical analysis on the disappearance rate of 13 individual symptoms of the modified Kupperman index in the Experimental group and the Placebo group at 12 weeks of treatment and 4 weeks after drug withdrawal (Table 3). Hot flashes and sweating, abnormal sensation, insomnia, irritability, urinary symptoms, painful intercourse (dyspareunia), depression, dizziness, fatigue, bone joint muscle pain, headache, palpitations formation in Experimental group at 12 weeks of treatment and 4 weeks after drug withdrawal The disappearance rate is significantly better than that of Placebo group (*p* < 0.001). The disappearance rate of 13 individual symptoms in the Experimental group exceeded 35% at 12 weeks of treatment and 4 weeks after discontinuation of treatment, while the disappearance rate in the vast majority of Placebo group groups did not exceed 25%. The disappearance rate of Formation and Depression in the Experimental group exceeds 60%, while the disappearance rate of Formation and Depression in the Placebo group is about 35%.

**TABLE 3:** Comparison of the disappearance rate of 13 individual symptoms of patients’ modified Kupperman index in the FAS.

Single symptom	Time	Experimental group	Placebo group	MD (95% CI)	*p value*
		Disappearance rate (%)	Disappearance rate (%)		
Hot flashes and sweating	12 weeks of treatment	30.51	3.39	0.27 (0.20, 0.33)	<0.0001***
	4 weeks of drug withdrawal	24.29	2.54	0.22 (0.15, 0.27)	
Abnormal sensation	12 weeks of treatment	50.81	11.65	0.39 (0.30, 0.47)	<0.0001***
	4 weeks of drug withdrawal	47.90	12.62	0.35 (0.26, 0.43)	
Insomnia	12 weeks of treatment	48.40	7.83	0.41 (0.32, 0.47)	<0.0001***
	4 weeks of drug withdrawal	41.98	6.09	0.36 (0.28, 0.42)	
Irritability	12 weeks of treatment	39.48	6.09	0.33 (0.26, 0.39)	<0.0001***
	4 weeks of drug withdrawal	41.50	1.74	0.40 (0.33, 0.45)	
Urinary symptoms	12 weeks of treatment	56.64	27.63	0.29 (0.16, 0.40)	<0.0001***
	4 weeks of drug withdrawal	57.08	30.26	0.27 (0.14, 0.38)	
Painful intercourse (dyspareunia)	12 weeks of treatment	50.95	15.94	0.35 (0.23, 0.45)	<0.0001***
	4 weeks of drug withdrawal	46.19	24.64	0.22 (0.08, 0.33)	<0.001***
Depression	12 weeks of treatment	61.25	33.77	0.27 (0.14, 0.38)	<0.0001***
	4 weeks of drug withdrawal	61.34	38.96	0.22 (0.09, 0.34)	<0.001***
Dizziness	12 weeks of treatment	60.14	29.79	0.30 (0.19, 0.40)	<0.0001***
	4 weeks of drug withdrawal	54.64	24.47	0.30 (0.19, 0.40)	
Fatigue	12 weeks of treatment	37.69	12.15	0.26 (0.16, 0.33)	<0.0001***
	4 weeks of drug withdrawal	37.39	9.35	0.28 (0.19, 0.35)	
Bone joint muscle pain	12 weeks of treatment	52.43	20.39	0.32 (0.22, 0.41)	<0.0001***
	4 weeks of drug withdrawal	53.72	20.39	0.33 (0.23, 0.42)	
Headache	12 weeks of treatment	60.08	33.72	0.26 (0.14, 0.37)	<0.0001***
	4 weeks of drug withdrawal	59.70	32.56	0.27 (0.15, 0.38)	
Palpitations	12 weeks of treatment	56.79	29.35	0.27 (0.16, 0.38)	<0.0001***
	4 weeks of drug withdrawal	52.86	19.57	0.33 (0.22, 0.42)	
Formication	12 weeks of treatment	66.98	40.30	0.27 (0.13, 0.39)	<0.0001***
	4 weeks of drug withdrawal	66.51	32.84	0.34 (0.20, 0.45)	

Abbreviations: FAS, full analysis set; MD, mean difference; CI, confidence interval. * represents *p* < 0.001.

### 3.4 Safety profile and adverse events

This study also conducted a statistical analysis of the AEs of all subjects (Table 4). Common adverse reactions of this drug are nausea, vomiting, diarrhea, stomach discomfort, allergic reactions (allergic rashes), palpitations, arrhythmias, high or low blood pressure, dizziness, headache, serious adverse reactions include abnormal liver function, abnormal kidney function, and abnormal blood system. A total of 149 AE were found in 123 subjects in the experimental group and placebo group, including 93 patients (26.2%) in the experimental group 119 times and 30 patients (25.4%) in the placebo group 30 times, with no significant difference (*p* > 0.05). Statistical analysis of adverse events in the experimental group and placebo group showed that the common adverse events in the experimental group were vomit, nausea, headache, and palpitations recorded 14, 12, 12, and 12 times respectively. The common adverse reactions in the placebo group are dizziness, headache, and diarrhea which were recorded 6, 5, and 5 times respectively (Supplementary Table S3). Adverse drug reactions (ADR) occurred in 24 cases and 28 times in the experimental group and placebo group, including 17 cases (4.8%) and 19 cases in the experimental group and 7 cases (5.9%) and 9 cases in the placebo group. Two serious adverse events (SAE) occurred in the experimental group and were blood system abnormalities, and both were judged not to be related to the therapeutic intervention of this study. The more common ADRs in the two groups were mild increases in urine microalbumin, glutamate aminotransferase and γ-glutamyl transpeptidase. And the correlation analysis results showed that the difference in ADR and SAE between experimental group and placebo group was not statistically significant (*p* > 0.05).

**TABLE 4:** Statistical analysis of adverse events in the SS.

Classification of AE	Experimental group (n = 355)		Placebo group (n = 118)		*p value*
	Number of patients (%)	Number of cases	Number of patients (%)	Number of cases	
AE	93 (26.2)	119	30 (25.4)	30	1.0000
ADR	17 (4.8)	19	7 (5.9)	9	0.6044
SAE	2 (0.6)	2	0 (0.0)	0	1.0000

Abbreviations: SS, safety set; AE, adverse events; ADR, adverse drug reactions; SAE, serious adverse events.

### 3.5 Subject compliance

The researchers valuated the medication status of the patients in the experimental group and placebo group through questionnaires at each follow-up. 350 (98.31%) in the experimental group patients had good compliance at the fourth week of discontinuation, and 116 (97.48%) patients in the placebo group had good compliance after 4 weeks of discontinuation. The overall compliance of the two groups reached 98.11%, indicating that the subjects had a high degree of compliance with the treatment plan.

## 4 Discussion

Menopausal Syndrome is caused by a decline in estrogen levels due to declining ovarian function in women (Kim et al., 2015). These symptoms can have a wide range of effects on patients’ physical and emotional functioning (Santoro et al., 2015). The decrease in estrogen affects the serotonin system in the brain, and the dysfunction of the serotonin system is closely related to anxiety (Valdés-Sustaita et al., 2021). The effect of estrogen on the dopamine system is also important, and the dysfunction of the dopamine system is related to depression (Zachry et al., 2021). Anxiety and depression are common psychological problems in women with menopausal syndrome and have always been the focus of researchers. This is the first double-blinded, multi-center randomized clinical trial to date to verify the safety and effectiveness of Kunxinning granules, a traditional Chinese medicine preparation, in the treatment of menopausal syndrome. Compared with the placebo group, the experimental group had significantly lower modified Kupperman index from baseline to week 12 after treatment. This study found that Kunxinning granules have a therapeutic effect on patients with menopausal syndrome.

The medicinal ingredients in Kunxinning granules have been shown to be useful in the treatment of menopausal syndrome, such as *R. glutinosa* (Kim and Kim, 2022) and *A. membranaceus* (Lin et al., 2020). Preclinical animal test results show that Kunxinning granules can reduce the spontaneous activities of mice, enhance the sedative effect of subthreshold sodium pentobarbital on mice, increase the uterine and adrenal gland coefficients of castrated rats and reduce the damage of uterine tissue (Yang et al., 2006). Moreover, Kunxinning granules can improve the sex hormone levels in castrated rats and aged female rats, which can indicate that it may relieve symptoms of autonomic dysfunction such as hot flashes and night sweats by regulating estrogen balance. The results of the phase II clinical trial showed that the Kunxinning granules group was better than the placebo group in terms of the reduction of the modified Kupperman index from baseline and the reduction of individual menopausal symptom scores from baseline.

HRT is the main method for treating menopausal syndrome, but HRT will increase the risk of many diseases during treatment, such as cardiovascular disease, cancer, thrombosis, and gallbladder disease. Moreover, HRT is not suitable for all patients with menopausal syndrome, and other methods are used clinically to treat these patients, such as non-hormonal drugs, lifestyle interventions, and Chinese botanical drugs. Kunxinning granules are a traditional Chinese medicine made from a variety of natural substances, many of which have been used to treat menopausal syndrome (Portella et al., 2024; Zhou et al., 2021). Rehmanni is rich in bioactive substances, such as iridoid glycosides, phenylethanoid glycosides and flavonoids (Piątcza et al., 2020), which help regulate hormone levels in patients with menopausal syndrome, improve antioxidant and anti-inflammatory, neuroprotective and enhance immunity (Li et al., 2019; Wu et al., 2022). Curculiginis is rich in curculigoside has antioxidant, anti-inflammatory, immunity-enhancing and endocrine function improvement effects (Zhang et al., 2024). Curculigoside mainly affects the balance of hormone levels in the body and helps relieve menopausal symptoms caused by estrogen reduction (Shan et al., 2016). Red Peony is rich in paeoniflorin and quercetin, kaempferol and gallic acid. Red Peony can improve blood circulation and relieve physical discomfort caused by blood stasis (Chen et al., 2022). Red Peony has an analgesic effect and can relieve common symptoms of menopausal women (Jiao et al., 2022). Red Peony has a sedative effect that can helps relieve mood swings and insomnia (Shi et al., 2016).

Compared with the commonly used treatments for menopausal syndrome, the incidence of adverse reactions of Kunxinning Granules is at a lower level. Common side effects of HRT include breast tenderness, weight gain, and increased risk of thrombosis (Vigneswaran and Hamoda, 2022). Although phytoestrogen drugs (such as soy isoflavones) have fewer side effects, they are more sensitive to individual differences, and some patients may experience gastrointestinal discomfort or endocrine disorders (Pandozzi et al., 2022). The results of this study showed that although Kunxinning Granules may cause mild gastrointestinal discomfort, headaches and palpitations, they are generally well tolerated and no serious adverse reactions were observed. Therefore, Kunxinning Granules performs better in terms of safety and can be used as a potential treatment option for patients with menopausal syndrome.

In this study, we selected female patients with menopausal syndrome aged 45-55 years as the research subjects. A key aspect of our Kun Mind Pellets is their safety. This study showed that the incidence of adverse events was similar between the experimental group and the placebo group. And serious adverse events were not related to therapeutic intervention. These results indicate that Kunxinning granules exhibit excellent safety in the treatment of menopausal syndrome.

However, the study design also has some limitations. This trial was conducted in nine hospitals in China. The modified Kupperman index of Kunxinning granules during the treatment phase of this study was significantly lower than that at baseline, but it is uncertain whether these findings can be extrapolated to different regions or populations. This study also needs to further determine the optimal treatment dose and treatment time in order to better evaluate the clinical efficacy of Kunxinning granules.

## 5 Conclusion

Kunxinning granules can effectively improve hot flashes and sweating, insomnia, irritability, urinary symptoms, and depression symptoms in patients with menopausal syndrome. On the basis of the efficacy and safety profiles, Kunxinning granules may be an effective alternative to HRT in the treatment of menopausal syndrome.

## Data Availability

The original contributions presented in the study are included in the article/Supplementary Material, further inquiries can be directed to the corresponding authors.

## Abbreviations

ADR, adverse drug reactions; AE, Adverse events; CI, confidence interval; FAS, full analysis set; FSH, follicle-stimulating hormone; MD, mean difference; PPS, per protocol pet; SAE, serious adverse events; SD, standard deviation; SS, Safety Set.
