# Supplementary figures and images for: Efficacy and safety of Kunxinning granules in patients with menopausal syndrome: a multicenter, randomized, double-blinded, and placebo-controlled trial
Source: Front Pharmacol. 2025 Jul 10;16:1512110. doi: 10.3389/fphar.2025.1512110 (PMC12287090; doi:10.3389/fphar.2025.1512110)

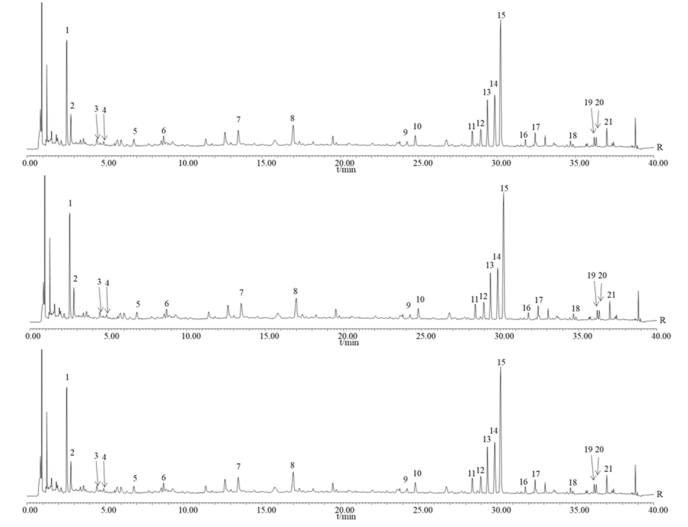

Supplement: Supplementary file 3 [file Image1.tif]
